# Peptidylarginine Deiminases Post-Translationally Deiminate Prohibitin and Modulate Extracellular Vesicle Release and MicroRNAs in Glioblastoma Multiforme

**DOI:** 10.3390/ijms20010103

**Published:** 2018-12-28

**Authors:** Uchini S. Kosgodage, Pinar Uysal-Onganer, Amy MacLatchy, Igor Kraev, Nicholas P. Chatterton, Anthony P. Nicholas, Jameel M. Inal, Sigrun Lange

**Affiliations:** 1 Cellular and Molecular Immunology Research Centre, School of Human Sciences, London Metropolitan University, London N7 8DB, UK; uchini22@hotmail.co.uk; 2 Cancer Research Group, School of Life Sciences, University of Westminster, London W1W 6UW, UK; P.onganer@westminster.ac.uk; 3 Research Centre for Optimal Health, School of Life Sciences, University of Westminster, London W1W 6UW, UK; amy.maclatchy@my.westminster.ac.uk; 4 School of Life, Health and Chemical Sciences, The Open University, Walton Hall, Milton Keynes MK7 6AA, UK; igor.kraev@open.ac.uk (I.K.); nicholas.chatterton@open.ac.uk (N.P.C.); 5 Department of Neurology, University of Alabama at Birmingham, Birmingham, AL 35294, USA; anicholas@uabmc.edu; 6 Extracellular Vesicle Research Unit and Bioscience Research Group, School of Life and Medical Sciences, University of Hertfordshire, Hatfield AL10 9AB, UK; j.inal@herts.ac.uk; 7 Tissue Architecture and Regeneration Research Group, School of Life Sciences, University of Westminster, London W1W 6UW, UK

**Keywords:** glioblastoma multiforme (GBM), peptidylarginine deiminase (PAD), extracellular vesicles (EVs), prohibitin (PHB), deiminated histone H3

## Abstract

Glioblastoma multiforme (GBM) is the most aggressive form of adult primary malignant brain tumour with poor prognosis. Extracellular vesicles (EVs) are a key-mediator through which GBM cells promote a pro-oncogenic microenvironment. Peptidylarginine deiminases (PADs), which catalyze the post-translational protein deimination of target proteins, are implicated in cancer, including via EV modulation. Pan-PAD inhibitor Cl-amidine affected EV release from GBM cells, and EV related microRNA cargo, with reduced pro-oncogenic microRNA21 and increased anti-oncogenic microRNA126, also in combinatory treatment with the chemotherapeutic agent temozolomide (TMZ). The GBM cell lines under study, LN18 and LN229, differed in PAD2, PAD3 and PAD4 isozyme expression. Various cytoskeletal, nuclear and mitochondrial proteins were identified to be deiminated in GBM, including prohibitin (PHB), a key protein in mitochondrial integrity and also involved in chemo-resistance. Post-translational deimination of PHB, and PHB protein levels, were reduced after 1 h treatment with pan-PAD inhibitor Cl-amidine in GBM cells. Histone H3 deimination was also reduced following Cl-amidine treatment. Multifaceted roles for PADs on EV-mediated pathways, as well as deimination of mitochondrial, nuclear and invadopodia related proteins, highlight PADs as novel targets for modulating GBM tumour communication.

## 1. Introduction

Glioblastoma multiforme (GBM) is the most common and aggressive form of primary malignant brain tumour in adults, with poor prognosis as only 28.4% of patients survive one year and 3.4% survive to year five [1,2,3]. Extracellular vesicles (EVs) are lipid bilayer-enclosed structures, 30–1000 nm in diameter, released from cells, and are key-mediators for intra/inter-tumour communication through horizontal transfer of functional proteins and nucleic acids (mRNA, miRNA, lncRNA) [4,5,6], through which GBM cells can influence the surrounding microenvironment to promote tumour growth, angiogenesis, metabolism and invasion [7,8,9,10,11]. The regulation of EV biogenesis has received increasing attention as an interceptive strategy in cancer, both to sensitize cancer cells to chemotherapy and to limit tumour growth in vivo [12,13,14,15,16,17,18]. The peptidylarginine deiminase (PAD)-mediated pathway of EV biogenesis has recently been described as a significant contributor to EV release in a range of cancer cells [14,17,19,20].

While EVs have been recognized to play significant roles in GBM, PADs have hitherto received little attention. PADs are a calcium-dependent enzyme family involved in physiological and pathophysiological processes [21,22,23,24]. PADs cause irreversible post-translational protein deimination (citrullination) of protein arginine to citrulline, using oxygen from water and releasing nitrogen as ammonia. Each conversion of an arginine into a citrulline causes the loss of one positive charge, increasing hydrophobicity and modifying folding of target proteins, leading to structural and functional protein changes [21,22,23,24,25,26,27]. Post-translational deimination may also facilitate protein moonlighting, an evolutionary acquired phenomenon where proteins are allowed to exhibit more than one physiologically relevant function within one polypeptide chain [28,29]. Protein structures identified as being most prone to deimination are intrinsically disordered proteins and β-turns, while the position of the arginine within the protein is also of importance [22,26,30]. In mammals, five tissue-specific PAD isozymes have been identified [21], with PADs and post-translational deimination widely studied in the past few years in relation to various pathologies including autoimmune diseases, central nervous system (CNS) pathologies and cancer [14,19,21,22,23,24].

Studies of PADs in CNS-related cancers have so far been limited. In a study on grade IV GBM patient samples, an increase in cytoplasmic and nuclear deiminated proteins was observed, but protein candidates were not identified [31]. Increased PAD4 staining has been observed in undescribed astrocytomas [32], while upregulation of PAD2 and PAD3 via cAMP-PKA (cyclic adenosine monophosphate- protein kinase A) signaling has been shown in U251MG astrocytoma cells [33] and PAD-upregulation was shown in response to hypoxia in malignant gliomas [34]. The presence of glioma stem-like cells is related to the high recurrence rates of GBM tumours and to GBM resistance to standard therapy, consisting of surgical resection followed by radiotherapy in addition to concomitant and adjuvant chemotherapy with temozolomide (TMZ) [35,36]. This may be of considerable relevance as there are indications that GBM stem cells reside preferentially within the hypoxic core of the tumour mass [34], while PAD activation has been linked to hypoxia in the CNS [34,37,38] and to modulation of neuronal stem cell growth and death [39]. As PAD-inhibitors have previously proven to be effective regulators of EV release in a number of cancers, and to sensitize various cancer cells to chemotherapy [14,17], we set out to identify PAD-mediated pathways in pro-oncogenic communication in GBM.

Using immunoprecipitation and proteomic approaches, various deiminated candidates were identified in two GBM cell lines, LN18 and LN229. Furthermore, changes in deimination of histone H3 and prohibitin (PHB) were validated in response to treatment with PAD-inhibitor Cl-amidine, as well as in combination with chemotherapy using TMZ. Histone H3 deimination is well recognized in relation to various cancers [24] and was previously associated with PAD-mediated EV release in prostate cancer by our group [14]. Recent studies have highlighted multifaceted roles of PHB in cell apoptosis and survival as well as in cancer, including for mitochondrial function and integrity [40,41]. Crucially, mitochondria are central to cancer survival and progression, in particular due to their central role in calcium signal control, which is altered in cancer [42,43]. In addition, EV-release has recently been related to changes in PHB levels and changes in mitochondrial function in cancer cells [18]. Furthermore, increased PHB levels are linked to chemo-resistance in cancers [44,45], while post-translational modifications of PHB can facilitate PHB shuttling between organelles to execute a variety of functions, including in cancer [40]. Therefore changes in these two deimination candidates were further investigated in GBM cells in response to Cl-amidine and combinatory treatment with TMZ in this study.

Modulatory effects of EV regulators on EV biogenesis, as well as on EV cargo, are of pivotal importance. Thus, the effect of Cl-amidine on miR21, a pro-oncogenic microRNA known to be enriched in GBM derived EVs [46,47], and on miR126, which has been found to be elevated in GBM patients with better prognosis [48], was evaluated. The two GBM cell lines under study were chosen as an example of a chemosensitive (LN229) and chemoresistant (LN18) GBM cell line respectively according to their identification as such cell lines in previously published literature [49].

Multifaceted effects of PAD-modulation on GBM, revealed in this study, indicate their potential as therapeutic targets for affecting GMB communication within the microenvironment and the penumbra.

## 2. Results

### 2.1. Protein Analysis

#### 2.1.1. PAD Isozyme Detection and Total Protein Deimination in GBM Cells

**Figure 1:**
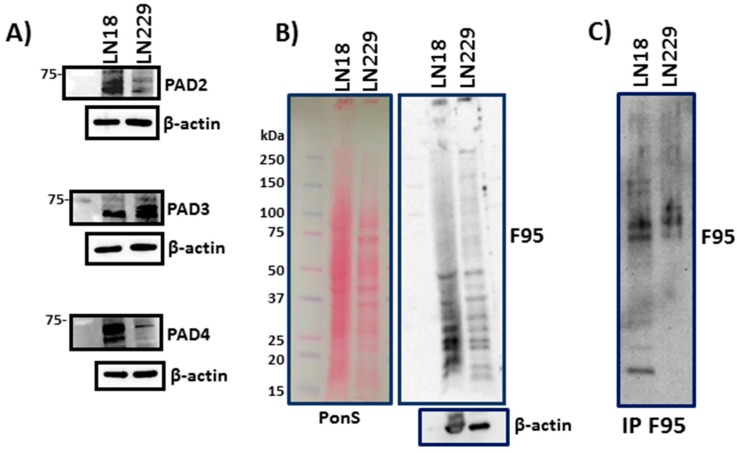
PADs and deiminated proteins in GBM cells. (**A**) PAD isozymes 2, 3 and 4 are detected at different levels in LN18 and LN229 GBM cells. (**B**) Western blotting showing total deiminated proteins (F95) in LN18 and LN229 cells (Ponceau S and β-actin shown as loading controls). (**C**) Immunoprecipitated deiminated proteins from both cell lines, using the F95 pan-deimination antibody for immunoprecipitation and detection.

Protein levels of PAD2, PAD3 and PAD4 isozymes differed in LN18 and LN229 cells; LN18 showed high levels of both PAD2 and PAD4, while LN229 had higher levels of PAD3 and low levels of PAD2 and PAD 4 (Figure 1A). Both LN18 and LN229 GBM cells showed considerable levels of deiminated proteins under normal culture conditions (Figure 1B). For identification of deiminated protein candidates, immunoprecipitated (F95 enriched) deiminated proteins from both cell lines (Figure 1C) were subjected to proteomic analysis (see Section 2.1.2 and Table S1).

#### 2.1.2. Liquid Chromatography-Tandem Mass Spectrometry (LC-MS/MS) Analysis of Deiminated Proteins in LN18 and LN229 GBM Cells

Deiminated protein eluates, immunoprecipitated from LN18 and LN229 cells using the pan-deimination F95 antibody [50], were separated by sodium dodecyl sulfate polyacrylamide gel electrophoresis (SDS-PAGE), and the extracted bands subjected to LC-MS/MS analysis with peak list files submitted to Mascot (Matrix Science, London, UK). Table S1 lists deiminated mitochondrial associated proteins identified both in LN18 and LN229 as well as nuclear-, stress-, EV-, cytoskeletal- and invadopodia-associated proteins. Changes in two deimination candidates, mitochondrial prohibitin (PHB) and nuclear histone H3, were further assessed in the presence of PAD-inhibitor Cl-amidine and TMZ by Western blotting (see Section 2.2 and Section 2.3).

**Figure 2:**
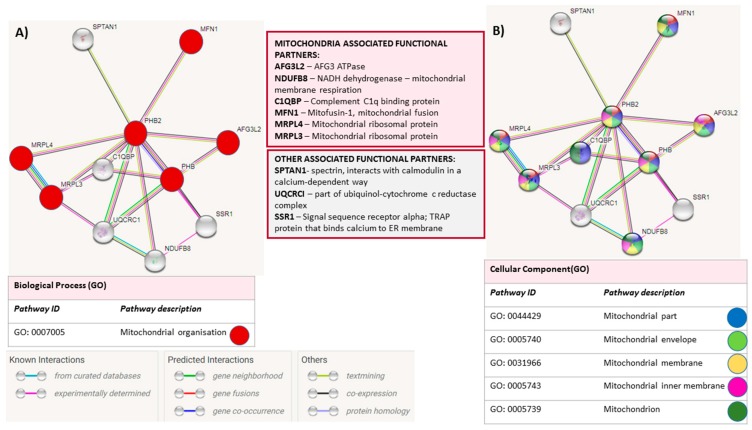
STRING analysis for Prohibitin. STRING analysis (https://string-db.org/) showing putative binding partners (STRING combined score >0.4) for prohibitin (PHB). Lines between nodes represent known interactions (from curated databases, blue; experimentally determined, pink), predicted interactions (gene neighbourhood, green; gene fusions, red; gene co-occurrence, blue) and other interactions (text-mining, lime green; co-expression, black; protein homology, grey). (**A**) Mitochondrial organisation associated functional partners are highlighted by red nodes; (**B**) Nodes are further colour coded for highlighting different mitochondrial parts (mitochondrial envelope and mitochondrial membrane).

STRING (Search Tool for the Retrieval of Interacting Genes/Proteins, https://string-db.org/) analysis for PHB is shown in Figure 2 with mitochondrial roles of identified binding partners highlighted.

#### 2.1.3. GBM Cell Viability in the Presence of Cl-Amidine and TMZ

LN18 cells showed a 15% decrease and 23% decrease in cell viability after 1 h incubation with 400 and 800 μM TMZ respectively, while the LN229 cells were not significantly affected with 800 μM TMZ (Figure S1A,B); thus 800 μM TMZ was the chosen working concentration. Cell viabilities of LN18 and LN229 cells were not significantly affected by Cl-amidine at (50 μM) (Figure S1C,D).

### 2.2. Prohibitin Protein and Post-Translational Deimination Levels Change after 1 h Cl-Amidine Treatment and in Combinatory Treatment with TMZ in GBM Cells

**Figure 3:**
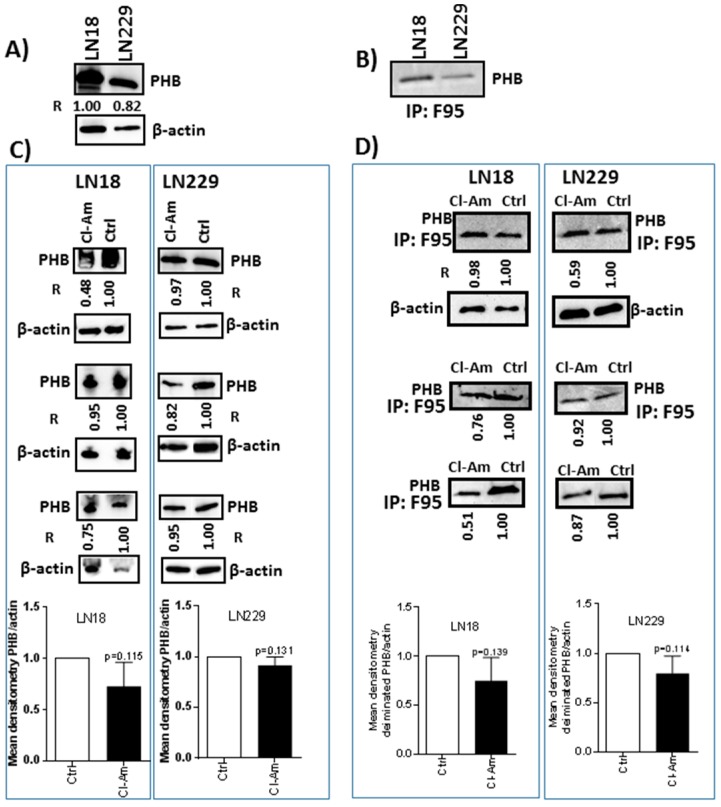
Prohibitin is post-translationally deiminated in GBM cells and affected by PAD-inhibitor Cl-amidine. (**A**) PHB protein is present in both GBM cell lines. (**B**) The presence of deiminated PHB is verified in both LN18 and LN229 cells, as assessed by probing the PHB antibody on F95-immunoprecipitated protein eluates (IP:F95). (**C**) PHB protein levels are reduced in LN18 and LN229 after 1 h treatment with Cl-amidine. (**D**) Post-translational deimination of PHB is reduced in GBM cells following 1 h Cl-amidine treatment. R represents relative densitometry compared to β-actin, which was used as the internal loading control.

**Figure 4:**
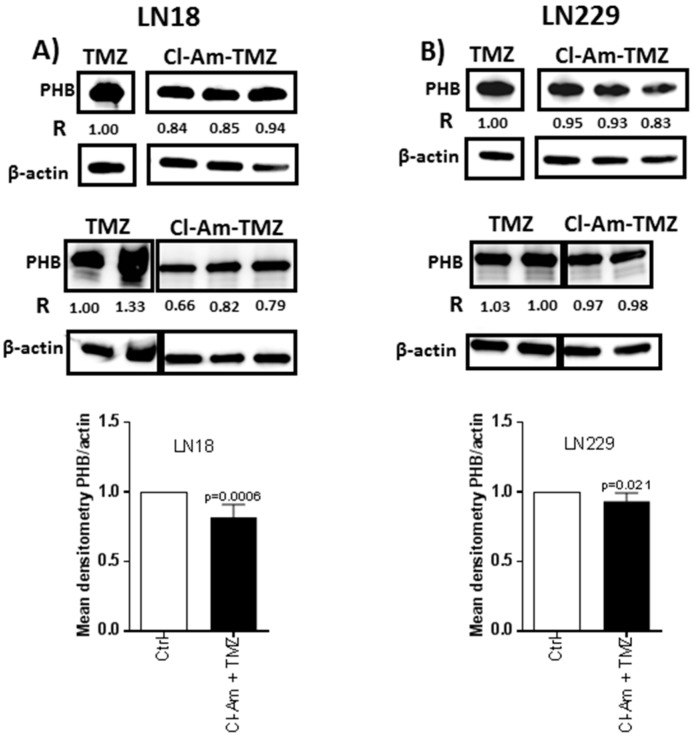
Cl-amidine treatment in combination with TMZ reduces PHB protein levels in GBM compared to TMZ treatment alone. (**A**) PHB protein levels in LN18 cells following 1 h combinatory Cl-amidine-TMZ treatment, compared to 1 h TMZ treatment alone. (**B**) PHB protein levels in LN229 cells following 1 h combinatory Cl-amidine-TMZ treatment compared to 1 h TMZ treatment alone.

The levels of PHB protein were higher in LN18 than LN229 cells (Figure 3A), and deiminated PHB was present in both cell lines (Figure 3B). After 1 h treatment with Cl-amidine, total PHB levels were reduced by 5–52% in LN18 and by 3–18% in LN229 cells (Figure 3C). After 1 h Cl-amidine treatment, deiminated PHB was reduced by 2–49% in LN18 cells and by 8–41% in LN229 cells (Figure 3D). After 1 h combinatory treatment of Cl-amidine with TMZ, PHB was reduced by 6–34% in LN18 cells, compared to TMZ treatment only (Figure 4A). LN229 GBM cells showed a 2–17% reduction in PHB following combinatory treatment versus TMZ alone (Figure 4B).

### 2.3. Deiminated Histone H3 Levels are Reduced after 1 h Cl-Amidine Treatment and Combinatory Treatment with TMZ in GBM Cells

**Figure 5:**
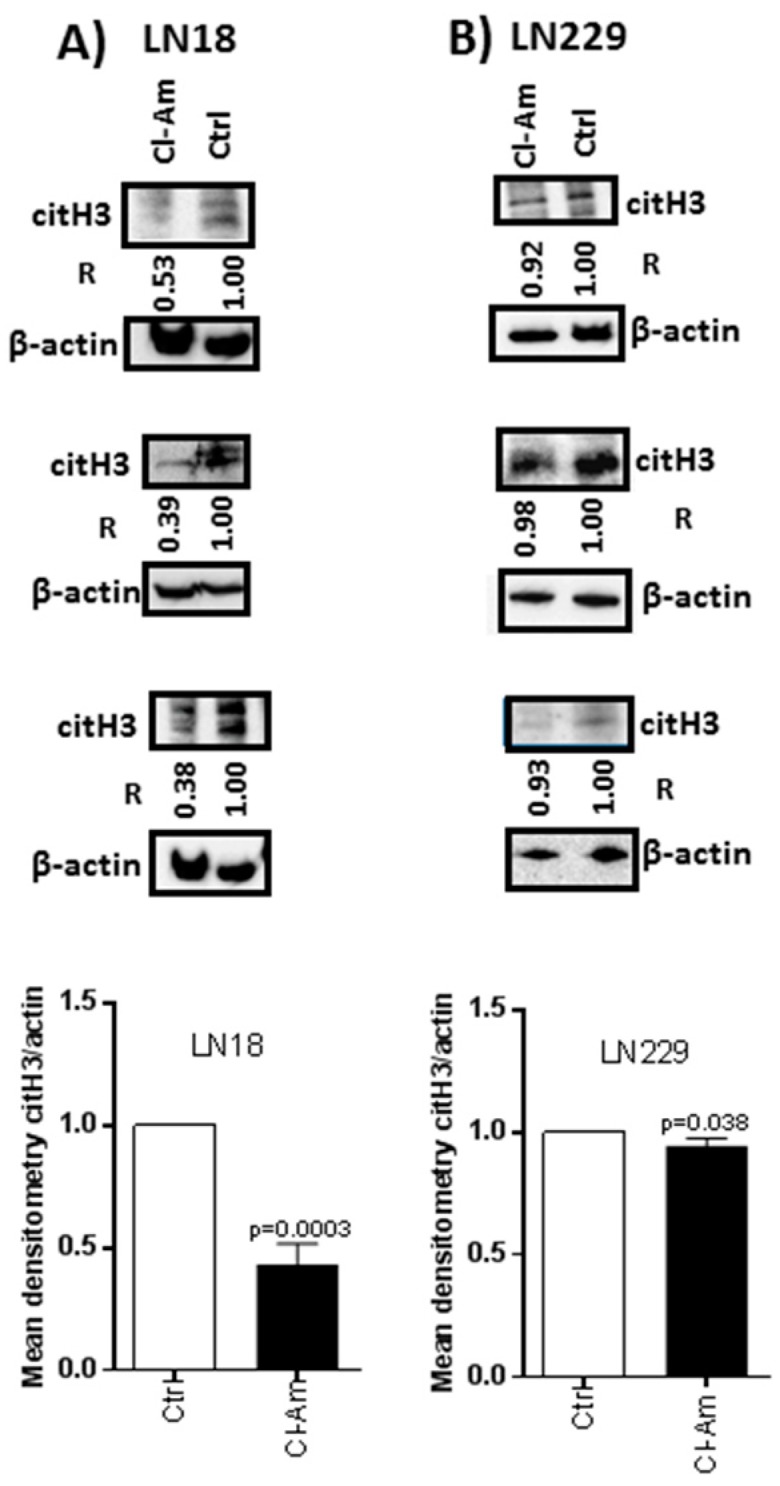
Deiminated histone H3 levels are reduced after 1 h treatment with PAD-inhibitor Cl-amidine. (**A**) Deimination of histone H3 (citH3) in LN18 cells following 1 h treatment with Cl-amidine. (**B**) Deimination of histone H3 (citH3) in LN229 cells after 1 h Cl-amidine treatment, compared to control untreated cells. R represents relative densitometry compared to β-actin, which was used as the internal loading control.

**Figure 6:**
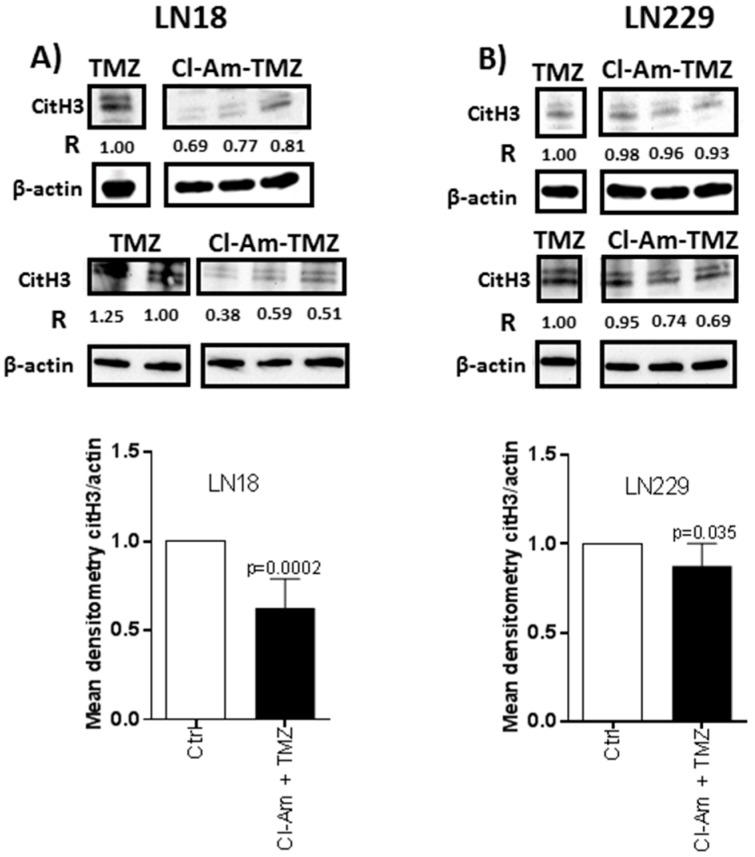
Cl-amidine treatment in combination with TMZ reduces citH3 levels in GBM compared to TMZ treatment alone. (**A**) Deiminated histone H3 (citH3) in LN18 cells following 1 h combinatory Cl-amidine-TMZ treatment, compared to 1 h TMZ treatment alone. (**B**) CitH3 in LN229 cells following 1 h combinatory Cl-amidine-TMZ treatment, compared to 1 h TMZ treatment alone. R represents relative densitometry compared to β-actin, which was used as the internal loading control.

Histone H3 deimination was reduced following 1 h Cl-amidine treatment by 47–62% in LN18 (Figure 5A) and 2–8% in LN229 cells (Figure 5B). After 1 h combinatory treatment with Cl-amidine and TMZ, citH3 levels were reduced by 19–62% in LN18 and 2–31% in LN229 cells, compared to TMZ treatment alone (Figure 6A,B).

### 2.4. Effects of Pan-PAD Inhibitor Cl-Amidine on EV Biogenesis in GBM Cells

**Figure 7:**
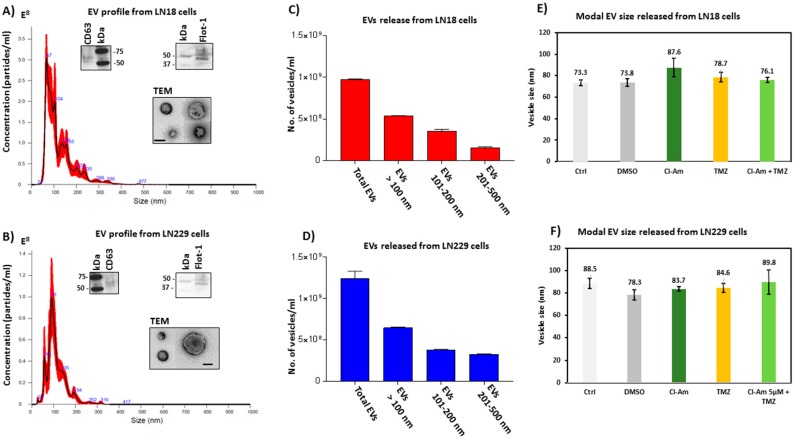
EV release in GBM cells under standard conditions and after 1 h Cl-amidine and TMZ treatment. (**A**) NTA histogram, as by Nanosight analysis, showing EVs released from LN18 GBM cells under standard conditions; EVs are characterized by EM and WB of EV-specific markers CD63 and Flot-1, as well as by TEM (scale bar is 100 nm). (**B**) NTA histogram showing EVs released from LN229 GBM cells under standard conditions; EVs are characterized by EM and WB of EV-specific markers CD63 and Flot-1, as well as by TEM (scale bar is 100 nm). (**C**,**D**) Proportions of EVs released from LN18 and LN229 cells under standard conditions. (**E**,**F**) Modal size of EVs released from LN18 and LN229 control and untreated cells, versus Cl-amidine or TMZ treated cells.

Both LN18 (Figure 7A,C) and LN229 (Figure 7B,D) cells showed a profile of EV release in the range of 20–500 nm. The EVs were characterized by electron microscopy and verified to be positive for the EV-specific markers CD63 and Flotillin-1 [51]. In LN18 cells, the modal size of EVs released (73.3 to 87.6 nm) did not differ significantly between treatment groups (Figure 7E), and the same was seen in LN229 cells (modal size of EVs 78.3 to 89.8 nm) (Figure 7F).

Further analysis of a numbers released of EVs in the size range ≤ 100 nm, EVs at 101–200 nm and EVs at 201–500 nm was performed between treatment groups, based on size-exclusion, using nanoparticle tracking analysis (NTA). After 1 h incubation with Cl-amidine (50 μM), LN18 cells showed 68.75% reduction of EVs ≤ 100 nm (*p* < 0.0001), 18.25% reduction (nil significance) in the 101–200 nm EVs, while a significant 76% increase (*p* = 0.0028) was observed in the 201–500 nm EVs, compared to control treated cells (Figure 7A,C,E). In LN229 cells, release of EVs ≤ 100 nm was reduced by 41.70% (*p* = 0.0074) after 1 h Cl-amidine treatment, while the 101–200 nm sized EVs were not significantly affected, but release of the 201–500 nm EVs was significantly reduced by 91.60% (*p* < 0.0001) (Figure 7B,D,F).

### 2.5. Effects of Cl-Amidine on EV Biogenesis in the Presence of TMZ

**Figure 8:**
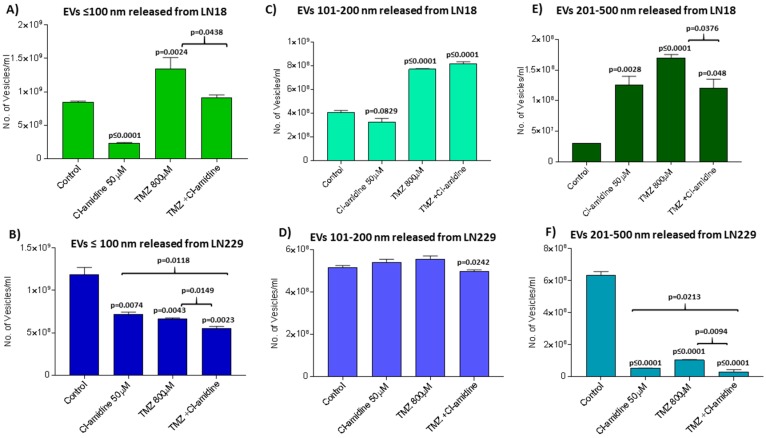
Cl-amidine, alone and in combination with TMZ, modulates EV release from GMB cells. EV release was assessed by NTA analysis after 1 h treatment with Cl-amidine, TMZ or TMZ in combination with Cl-amidine. (**A**) Release of EVs ≤ 100 in the LN18 GBM cell line after 1 h treatment. (**C**) Release of 101–200 nm EVs in LN18 cells following 1 h treatment. (**E**) Release of 201–500 nm EVs in LN18 cells following 1 h treatment. (**B**) Release of EVs ≤ 100 in LN229 cells following 1 h treatment. (**D**) Release of 101–200 nm EVs in LN229 cells following 1 h treatment. (**F**) Release of 201–500 nm EVs in LN229 cells following 1 h treatment. The *p*-values indicated above the bars in the histograms are significant changes compared to control treated cells; significant changes between TMZ and combinatory treatment of TMZ with Cl-amidine are also indicated by brackets.

For LN18 cells, 1 h incubation with TMZ increased release of EVs ≤ 100 nm by 62.50% (*p* = 0.0024) (Figure 8A), the 101–200 nm EVs by 49.37% (*p* < 0.0001) (Figure 8C) and the 201–500 nm EVs by 82.35% (*p* < 0.0001) (Figure 8E). For LN229 cells, 1 h incubation with TMZ decreased release of EVs ≤ 100 nm by 41.70% (*p* = 0.0043) (Figure 8B), did not significantly 101–200 nm sized EVs (Figure 8D) and reduced EVs in the 201–500 nm size range by 94.00% (*p* < 0.0001) (Figure 8F).

The two GBM cell lines also differed in EV release profiles after 1 h treatment with Cl-amidine and TMZ. For LN18 cells, the TMZ-induced release of EVs ≤ 100 nm was reduced by 31.20% in the combinatory treatment with Cl-amidine (*p* = 0.0438; Figure 8A), compared to TMZ alone. Combinatory Cl-amidine-TMZ incubation had no significant effect on TMZ-induced release of 101–200 nm sized EVs (Figure 8C), but combinatory treatment did reduce the TMZ induced release of 201–500 nm sized EVs by 29.41% (*p* = 0.0376; Figure 8E). In the LN229 GBM cells, Cl-amidine in combination with TMZ reduced the release of EVs ≤ 100 nm by 16.21% (*p* = 0.0149) compared to TMZ alone (Figure 8B), and that of the 101–200 nm EVs by 10.77% (*p* = 0.0242; Figure 8D) and also significantly reduced the release of EVs in the 201–500 nm range by 90.00% (*p* = 0.0094) compared to TMZ treatment alone (Figure 8F).

### 2.6. Cl-Amidine Modulates miRNAs in GBM Cells and Derived EVs

**Figure 9:**
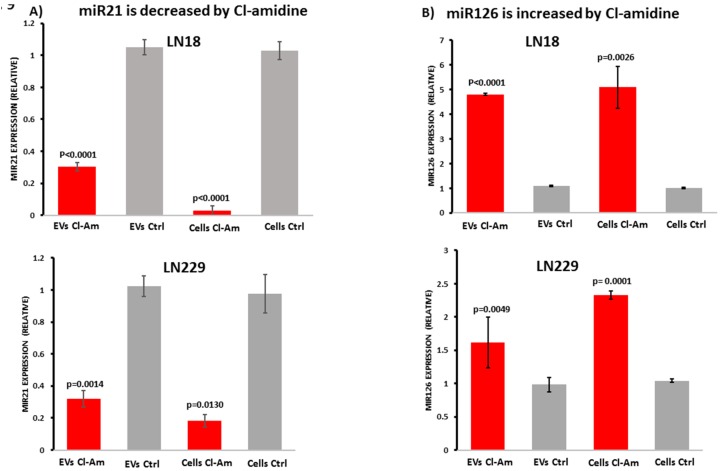
Cl-amidine reduces miR21 and increases miR126 export in EVs released from GBM cells. (**A**) Pro-oncogenic miR21 relative expression in EVs released from LN18 and LN229 GBM cells and the respective cell lysates. (**B**) Anti-oncogenic miR126 relative expression in cell lysates and in EVs released from both LN18 and LN229 cells. Exact *p*-values for changes in Cl-amidine versus control treated cells are indicated (*n* = 4 for each treatment group for LN18; *n* = 3 for each treatment group for LN229. Relative fold-changes are shown.

LN18 and LN229-derived EVs, and their respective cell lysates, were analysed for relative changes in microRNA expression [52] for the pro-oncogenic miR21 and the anti-oncogenic miR126 following 1 h incubation with Cl-amidine. Compared to un-treated control cells, relative expression of pro-oncogenic miR21 was significantly reduced both in LN18 and LN229-derived EVs and the respective cell lysates (Figure 9A). The levels of anti-oncogenic miR126 were significantly increased in both cell lysates and cell-derived EVs after 1 h treatment with Cl-amidine (Figure 9B).

### 2.7. Cl-Amidine in Combination with TMZ Modulates miRNAs in GBM Cells and Derived EVs

**Figure 10:**
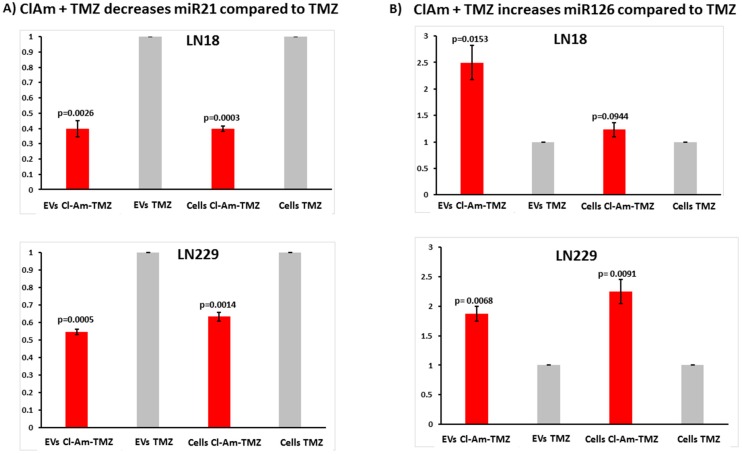
Cl-amidine in combination with TMZ reduces miR21 and increases miR126 export in EVs released from GBM cells, compared to TMZ alone. (**A**) Pro-oncogenic miR21 relative expression in EVs released from LN18 and LN229 GBM cells, and respective cell lysates. (**B**) Anti-GBM associated miR126 relative expression in cell lysates and EVs released from LN18 and LN229 cells. Exact *p*-values are indicated on the graph (*n* = 3 for each treatment group for LN18; *n* = 3 for each treatment group for LN229. Relative fold-changes are shown.

GBM cells were further assessed for modulation in microRNA cargo following 1 h treatment with TMZ alone versus combinatory treatment of TMZ with Cl-amidine (Figure 10A,B). After 1 h combinatory treatment, pro-oncogenic miR21 was significantly reduced both in EVs released from LN18 and LN229 GBM cells, as well as in the respective cell lysates, compared to TMZ treatment alone (Figure 10A). Anti-GBM associated miR126 was significantly increased after 1 h combinatory Cl-amidine-TMZ treatment in EVs released from both LN18 and LN229 cells, compared to TMZ treatment alone (Figure 10B). In the respective cell lysates, miR126 was significantly increased in LN229 cells, and while also increased in LN18 cells, the difference was not statistically significant compared to TMZ treatment alone (Figure 10B).

## 3. Discussion

The two GBM cell lines under study contained a range of deiminated proteins under normal culture conditions. In LN18 cells PAD2 and PAD4 were the dominating isozymes, while in LN229 cells PAD3 was the main isozyme. The three PAD isozymes have been described in the CNS and show distinct substrate preferences although some targets overlap [53,54,55,56,57,58]. All three isozymes have been detected in the nucleus, albeit PAD4 is the only isozyme with a classic nuclear translocation signal [39,59,60,61,62]. Both PAD2 and PAD4 have roles in various cancers via changes in cell proliferation, invasion and regulation of tumour growth [60,63,64,65,66], as well as affecting gene transcription and epigenetic cross talk [67]. PAD3 plays roles in CNS regeneration [61] and is associated with neuronal stem cells [39]. The strong presence of PAD3 in LN229 may thus indicate more stem-like properties of this GBM cell line. Some proteins previously identified as deiminated in hypoxic astrocytoma cell lysates [34] were here identified in both LN18 and LN229 under normal culture conditions, including cytoskeletal proteins (vimentin, filamin-A, cytoplasmic actin-1) and stress-related proteins (GRP78: 78 kDa glucose-regulated protein and G3P: Glyceraldehyde-3-phosphate dehydrogenase) (Table S1).

Cl-amidine [68] is a pan-PAD inhibitor, and although more recent and PAD-isozyme specific inhibitors have been developed [69,70,71,72,73,74,75,76,77,78,79,80,81], Cl-amidine was used here as a first proof of principle for PAD-modulation in GBM, particularly as Cl-amidine is an effective EV inhibitor in various cancer cells [14,17,19,20] and promotes CNS repair [37,38,61]. Deimination of histone H3 was found reduced both in LN18 than LN229 cells following Cl-amidine treatment. Prohibitin (PHB) levels were reduced in both cell lines following Cl-amidine treatment, as well as deiminated PHB, as verified by blotting the F95 immunoprecipitated eluate with the PHB antibody. While PHB has been linked to GBM regulation [82,83] and associated with high grade tumours [84,85,86], post-translational deimination of PHB has hitherto not been described. As central roles for post-translational modifications are increasingly acknowledged in the multifaceted functions of PHB [40,87], the newly discovered deimination of PHB here may be of considerable interest. Post-translational modifications facilitate shuttling of PHB between organelles [40], can affect each other [38], and furthermore, PHB can be regulated via micro-RNAs [88,89]. Accumulation of PHB is a common cellular response to stressing stimuli and can protect cancer cells from ER stress and chemotherapy-induced cell death [45]. Thus, reduced levels of PHB protein, as well as changes in deiminated PHB observed here after 1 h Cl-amidine treatment, may affect GBM functions including via mitochondrial function and changes in chemoresistance.

Further deiminated proteins identified and related to GBM invasion and progression included AHNAK (Neuroblast differentiation-associated protein; Table S1), which was identified in LN18 cells only. AHNAK has been shown to enable EV release from mammary carcinoma cells, playing critical roles in EV communication for promotion of cancer progression in the tumour microenvironment [90]. Stromal interacting molecule 1 (STIM1), was identified as deiminated in both LN18 and LN229 cells, and is a membrane ER-resident protein and an ER Ca^2+^ sensor, involved in sustaining long-term calcium signaling and thus critical for cellular functions [91,92]. STIM1 protein has been found to be elevated in several human cancer cells including in GBM where STIM activity is essential for invasion [93] while STIM silencing shows anti-proliferative effects both in vitro and in vivo [94]. Moesin was identified as deiminated in LN18 cells only; it connects the actin cytoskeleton to transmembrane receptors and increases cell invasion and migration of various GBM cells upon upregulation [95]. Moesin acts as an oncogene by increasing stem cell neurosphere formation and its overexpression is related to more aggressive and high-grade GBM [96,97]. Phosphorylation of moesin has been shown to be involved in its activation and interaction with CD44 and the Wnt/β-catenin pathway [96], but post-translational deimination of moesin has not been described before. The deiminated candidate proteins identified here (Table S1), were further matched against previously identified key invasive proteins which were also found exported in EVs from six GBM cell lines [98]. Out of fourteen proteins identified as markers for more aggressive disease four were here identified as deimination candidates; Cathepsin D and GAPDH were common to both LN18 and LN229, while Annexin A1 and Integrin beta-1 were identified as deiminated in LN229 only (Table S1). 

Histones undergo various posttranslational modifications that affect gene regulation and can also act in concert [99,100]. Histones known to undergo deimination are H2A [101], H2B [102], H3 and H4 [103] and were here identified as being deiminated in GBM cells (Table S1). In addition, HDAC (histone deacetylase) 1 and 2, histone-binding protein and histone H1x were identified as deiminated in GBM cells (Table S1). Therapeutic targeting of histone modifications has received considerable attention in high-grade gliomas, where HDAC overexpression has been reported in high-grade, late-stage proliferative tumors [104]. Recent findings also support roles for histone H3 deimination/methylation cross-talk via PAD2 and PAD4 in the regulation of gene transcription in cancer [105], while crosstalk between histone deacetylation and deimination via PAD4 and HDAC2 regulates p53 [67]. Furthermore, PAD4 has also been shown to interact with HDAC1 in vitro and in vivo [106]. Histone deimination has also been shown to regulate miRNA expression and oncogenic mRNAs [107] and identified as a driver of Interleukin-6 production [108]. In addition, deiminated histone H3 is a key player in neutrophil release of nuclear chromatin and a marker of neutrophil extracellular trap formation, which is related to promoted tumour progression and spread, including in GBM [109].

Effects of PAD inhibitor Cl-amidine on microRNA expression, both in GBM cells and derived EVs, highlights approaches for targeted modulation of EV cargo to change GBM intra- and inter-tumour communication. Chemotherapy with TMZ has previously been shown to affect EVs released by GBM cells [110], and here we found changes both in EV numbers and microRNA cargo released upon combinatory treatment with Cl-amidine and TMZ, compared to TMZ treatment alone. Notably we observed a higher sensitivity of LN18 to 1 h treatment with high doses of TMZ than seen in LN229, which somewhat contradicts with previous literature indicating that LN18 is a chemoresistant GBM cell line [49]. It must though be considered that such observed chemoresistance was following repeated TMZ exposure at lower levels of TMZ for longer time periods [49] and this may thus not be reflected in the 1 h high dose treatment performed in the current study. Furthermore, LN18 showed higher levels of EV release in the presence of TMZ, which may correlate with previously published studies on chemoresistance of LN18, and may imply that increased EV release acts as a mechanisms to facilitate drug efflux, as has been shown for other cancers [12,13,15,16]. In addition, in the LN18 cells an increase in release of 201–500 nm sized EVs in response to Cl-amidine treatment observed here, may indicate signs of pseudoapoptotic responses, where the cell can use the apoptosome to form an EV to export hazardous agents [111,112].

Changes in microRNA21 have been shown to affect viability, senescence and invasion in GBM [47,113], with miR21 silencing leading to decreased tumour size and improved survival in GBM animal models [114]. Inhibition of miR21 has also been shown to enhance chemo-sensitivity of TMZ-resistant GBM cells in vitro [115]. In GBM-derived patient samples, miR126 is significantly lower than in paired non-tumoural controls and related to high histopathological grades; while patients with higher intra-tumoural miR126 levels have significantly improved survival duration compared to patients with lower miR126 levels [48]. In vitro, over-expression of miR126 suppresses glioma cell proliferation and invasion via regulation of ERK (extracellular signal-regulated kinase) and KRAS (Kirsten rat sarcoma viral oncogene) [116]. The observed decrease in pro-oncogenic miR21 and increase in anti-oncogenic miR126 levels, caused by Cl-amidine here, indicates thus active anti-GBM functions of this PAD-inhibitor.

Various proteins, critical to GBM progression, were identified to be deiminated while some differences were observed between LN18 and LN229 cells. This may possibly reflect preferences in target proteins deiminated by the different PAD isozymes, further emphasising GBM heterogeneity. Besides PAD-enzymes being calcium-regulated themselves, their downstream deimination of proteins involved in calcium regulation as identified here, such as STIM and proteins crucial for mitochondrial integrity, given also that the mitochondrium has a central role in calcium homeostasis [42,43,117,118], indicate a complex involvement in EV biogenesis, which itself is driven by calcium [119,120,121]. Nonetheless, the observed ability of Cl-amidine to modulate EVs and associated microRNA cargo, as well as affecting PHB and histone H3 deimination, indicates common PAD-mediated pathways. Using tailored PAD-inhibitors may thus offer novel strategies for GBM cancer treatment and sensitization to chemotherapy.

## 4. Materials and Methods

### 4.1. Cell Cultures—LN18 and LN229

LN18 (ATCC^®^ CRL-2610™, grade IV glioblastoma derived from a male patient with a right temporal lobe glioma) and LN229 (ATCC^®^ CRL-2611™, glioblastoma derived from a female patient with right frontal parietal-occipital glioblastoma) (American Type Culture Collection, Manassas, VA 20108, USA), were cultured using ATCC’s recommendations to 80% confluence in 75 cm^2^ flasks in complete Dulbecco’s Modified Eagle’s Medium (DMEM Gibco®, ThermoFisher Scientific, Loughborough, Leicestershire, UK), with 5% foetal bovine serum (FBS Gibco®, ThermoFisher Scientific, UK) at 37 °C/5% CO_2_. The cell lines were chosen as an example of a chemo-resistant (LN18) and chemo-sensitive (LN229) GBM cell line respectively, according to previously published literature [49].

### 4.2. Protein Analysis of GBM Cells

#### 4.2.1. Protein Preparation

Total protein was extracted from LN18 and LN229 cells, or isolated EV pellets, in the presence of RIPA+ buffer (Sigma-Aldrich, Saint Louis, MO 63103, USA) containing 10% protease inhibitor complex (Sigma-Aldrich), pipetting gently with regular intervals while shaking the cell preparation on ice for 2 h. Thereafter the cell or EV preparations were centrifuged at 16,000× *g* (4 °C/20 min) and the supernatant containing the extracted protein collected. Protein extracts were either used immediately for immunoprecipitation and proteomic analysis, or re-constituted in 2× Laemmli sample buffer for Western blotting.

#### 4.2.2. Immunoprecipitation and Proteomic Analysis of Deiminated Protein Candidates from LN18 and LN229 GBM Cell Lines

For isolation of total deiminated proteins from LN18 and LN229 cells, the monoclonal F95 pan-deimination antibody, that is raised against a deca-citrullinated peptide and specifically detects protein citrulline [50], was used in conjunction with the Catch and Release^®^ v2.0 Reversible Immunoprecipitation System (Merck, Nottingham, UK), according to the manufacturer’s instructions. Bound proteins were eluted and either subjected to Western blotting analysis after re-constitution in 2× Laemmli sample buffer, or analysed by LC-MS/MS for identification of deiminated protein candidates, with the peak list files submitted to MASCOT (Cambridge Centre for Proteomics, Cambridge, UK).

#### 4.2.3. Western Blotting Analysis

Protein extracts from LN18 and LN229 cells or EVs (as described in 4.2.1), in 2× Laemmli sample buffer containing 5% β-mercaptoethanol, were boiled for 5 min at 100 °C before separation by SDS-PAGE, using 4–20% Mini-Protean TGX protein gels (BioRad, Watford, UK), followed by Western blotting analysis. Approximately 5 μg of protein was loaded per lane and even transfer to nitrocellulose membranes (0.45 μm, BioRad) was assessed using Ponceau S staining (Sigma-Aldrich). The membranes were blocked for 1 h at room temperature (RT) in 5% BSA (Sigma-Aldrich) in Tris buffered saline (TBS) with 0.001% Tween20 (TBS-T), followed by overnight incubation at 4 °C with the following primary antibodies for the cell lysates (used 1/2000 in TBS-T): anti-PAD2 (ab50257, Abcam, Cambridge, UK), anti-PAD3 (ab50246), anti-PAD4 (ab50332), anti-prohibitin (ab75771), anti-citH3-r2-r8-r17 (ab5103), F95 (1/5000; [45]), while for EV characterization the EV-specific markers CD63 (ab68418) and Flot-1 (ab41927) were used (1/1000 in TBS-T). Thereafter, membranes were washed in TBS-T, incubated for 1 h at RT with the corresponding HRP-conjugated secondary antibodies (anti-rabbit IgG or anti-mouse IgM, BioRad), followed by TBS-T washes and visualisation using ECL (Amersham, Fisher Scientific, Loughborough, Leicestershire, UK) and the UVP BioDoc-ITTM System (Fisher Scientific, Loughborough, Leicestershire, UK). HRP-conjugated anti-β-actin antibody (ab20272, Abcam, 1/5000 in TBS-T) was used as an internal loading control and densitometry analysis was carried out using ImageJ1 (https://imagej.net/ImageJ1).

### 4.3. Cell Viability Assays

Cell viability of LN18 and LN229 GBM cells was assessed after 1 h incubation with Cl-amidine (50 μM, a kind gift from Prof Paul Thompson, UMASS) and after 1 h incubation with TMZ (Sigma-Aldrich) concentrations of 100, 200, 400 or 800 μM, compared to DMSO control-treated cells (Figure S1A,B). Cell viability was further assessed after 1 h combinatory treatment of Cl-amidine (50 μM) with TMZ (800 μM), compared to Cl-amidine alone (50 μM), TMZ alone (800 μM) or control-treated cells (Figure S1C,D). Cell viability assessment was carried out before the start of every experiment using the Guava ViaCount cell death assay (Guava Millipore) as previously described [17,18].

### 4.4. Modulation of EV Biogenesis Using Pan-PAD Inhibitor Cl-Amidine

The effect of Cl-amidine (50 μM) on EV release after 1 h incubation was assessed. LN18 and LN229 cells were seeded at a density of 5 × 10^5^ cells per well, in triplicate, in the presence of culture medium (pre-warmed DMEM, supplemented with 10% FBS; Gibco®, ThermoFisher Scientific, UK). The cell preparations were thereafter washed with pre-warmed PBS (EV-free), and resuspended in pre-warmed serum- and EV-free DMEM and plated at 5 × 10^5^ cells per well. Cl-amidine (50 μM) in PBS was incubated with the cells for 1 h at 37 °C/5% CO_2_; PBS (EV-free) treated cells were used as controls. The plates were briefly placed on ice (1 min) and the supernatant collected from each well. Cell debris was removed by centrifugation at 200× *g* for 5 min and thereafter EVs were isolated from the remaining supernatant as described in 4.6.

### 4.5. Effects on EV Biogenesis in the Presence of Temozolamide (TMZ)

LN18 and LN229 cells were cultured and prepared for EV isolation and quantification as described in 2.4 and respectively treated for 1 h with 50 μM Cl-amidine alone as before, for 1 h with TMZ alone (800 μM in 0.001% DMSO as determined by the cell viability assay in 4.3) or for 1 h with a combination of Cl-amidine (50 μM) and TMZ (800 μM). DMSO-treated cells were used as control.

### 4.6. EV Isolation and Quantification by Nanoparticle Tracking Analysis–1 h Treatment

Differential centrifugation was carried out on the cell culture supernatants as follows: First the supernatants were centrifuged at 4000× *g* for 1 h for removal of cell debris, followed by centrifuging the collected supernatant at 100,000× *g* for 1 h/4 °C. The isolated EV pellets were then resuspended and washed in Dulbecco’s PBS (DPBS), centrifuged at 100,000× *g* for 1 h/4 °C and thereafter resuspended in 100 μL sterile EV-free PBS. Nanoparticle tracking analysis (NTA) was carried out using the NS300 Nanosight (Malvern Panalytical, Malvern, Worcestershire, UK), equipped with a sCMOS camera and a 405 nm diode laser, to enumerate the EVs. Samples were diluted 1:10 in sterile-filtered EV-free DPBS and the number of particles in the field of view was maintained in the rage of 20–40 with a minimum concentration of samples at 5 × 10^7^ particles/mL. Camera settings were according to the manufacturer’s instructions (Malvern Panalytical), recording four 90 s videos per sample and averaging the obtained replicate histograms. Each experiment was repeated three times.

### 4.7. Preparation of EVs for Transmission Electron Microscopy (TEM)

A suspension of isolated EVs was fixed with 2.5% glutaraldehyde in 100 mM sodium cacodylate buffer (pH 7.0) for 1 h at 4 °C, whereafter they were gently pelleted, washed and re-suspended in 100 mM sodium cacodylate buffer (pH 7.0). Next, a drop (~5–10 μL) of the suspension was placed on to a grid with carbon support film previously glow discharged. When the suspension had partly dried, the grid was washed by touching it three times to the surface of a drop of distilled water. Excess water was removed by touching the grid to a filter paper. A small drop of stain (2% aqueous Uranyl Acetate, Sigma-Aldrich) was then applied to the grid. After 10 s the excess stain was removed by touching the edge to a filter paper. The grid was dried at room temperature and thereafter the samples were viewed in TEM.

### 4.8. miRNA Analysis in GBM Cells and Derived EVs

For assessment of microRNA cargo in the GBM-derived EVs, LN18 and LN229 cells were cultured to 75% confluency in T75 flasks in DMEM supplemented with 10% FBS. The cells were washed with EV-free DPBS and thereafter fresh EV and serum-free medium was added, containing Cl-amidine (50 μM), TMZ (800 μM) or a combination of TMZ (800 μM) and Cl-amidine (50 μM), and 0.001% DMSO for control treatment. After 1 h incubation time, the cell medium was collected for EV isolation and the cells were pelleted for further RNA isolation and microRNA analysis. EVs were isolated as described in 4.6 for RNA isolation, cDNA translation and assessment for expression of microRNAs miR21 and miR126. Each experiment was repeated three times. RNA was extracted from treated and non-treated control cells using Trizol (Sigma-Aldrich) and RNA concentration and purity was measured using the NanoDrop Spectrophotometer at 260 and 280 nm absorbance. RNA was reverse transcribed to cDNA using the qScript microRNA cDNA Synthesis Kit (Quantabio, Beverly, MA 01915, USA) according to the manufacturer’s protocol. The resulting cDNA was used to assess the expression of microRNAs miR21, the main microRNA associated with pro-oncogenic function, and miR126, associated with protective function in GBM, while U6 was used as a reference RNA for normalisation of miR expression levels. The PerfeCTa SYBR^®^ Green SuperMix (Quantabiowas used together with MystiCq microRNA qPCR primers for both miR21 (hsa-miR-21-5p) and mir126 (hsa-miR-126-5p), which were obtained from Sigma-Aldrich). The sequences for U6 primers were U6 forward, 5′-GCTTCGGCAGCACATATACTAAAAT-3′ and reverse 5′-CGCTTCACGAATTTGCGTGTCAT-3′. The thermocycling conditions were as follows: Denaturation at 95 °C/2 min, followed by 40 cycles at 95 °C/2 s and 60 °C/15 s, and extension at 72° C/15 s. The miR21 and miR126 expression levels were normalized to that of U6 using the 2(−Delta Delta *C*(T)) method according to Livak and Schmittgen [52].

### 4.9. Statistical Analysis

The histograms and graphs were prepared and statistical analysis was performed using GraphPad Prism version 6 (GraphPad Software, San Diego, CA, USA). One-way ANOVA was performed followed by Tukey’s post-hoc analysis. Experiments were repeated in triplicates, histograms represent mean of data and standard error of mean (SEM) are indicated by the error bars. Significant differences were considered as *p* ≤ 0.05.

## 5. Conclusions

Here we show novel roles for PAD-mediated deimination in two GBM cell lines, including the identification of mitochondrial, nuclear and invadopodia-related protein targets. For the first time a modulatory effect of pan-PAD-inhibitor Cl-amidine is shown on EV release and EV cargo in GBM cells. Cl-amidine treatment resulted in reduction of pro-oncogenic miR21 and elevation of anti-oncogenic miR126 in GBM cells and derived EVs, both when used alone or in combination with TMZ, the standard chemotherapeutic drug for GBM. The two GBM cell lines under study varied in PAD isozymes, indicating PAD-mediated contribution to GBM heterogeneity. Histone H3 deimination was found to be reduced in GBM following Cl-amidine treatment. Furthermore, prohibitin (PHB), a multifaceted protein involved in mitochondrial housekeeping and cancer chemo-resistance, was identified for the first time to be post-translationally deiminated in GBM cells and reduced upon Cl-amidine treatment. Our findings indicate that PAD-inhibition may be used to lower anti-chemotherapeutic responses of GBM to TMZ and to modulate EV-mediated communication of GBM, both by affecting EV numbers released and by modifying EV cargo to an anti-oncogenic signature.

## Supplementary Materials

Supplementary materials can be found at http://www.mdpi.com/1422-0067/20/1/103/s1. **Table S1**: Deiminated protein candidates identified by LC-MS/LS in LN18 and LN229 cells, **Figure S1**: GBM cell viability after 1 h incubation with Cl-amidine, TMZ and Cl-amidine in combination with TMZ.

## Abbreviations

AHNAK	Neuroblast differentiation-associated protein
citH3	Deiminated Histone H3
CNS	Central Nervous System
DMEM	Dulbecco’s Modified Eagle’s Medium
EVs	Extracellular Vesicles
ER	Endoplasmic Reticulum
FBS	Foetal Bovine Serum
GBM	Glioblastoma Multiforme
G3P	Glyceraldehyde-3-phosphate dehydrogenase
GRP78	78 kDa glucose-regulated protein
HDAC	Histone Deacetylase
LC-MS/MS	Liquid Chromatography Mass Spectrometry
miRNA	microRNA
MV	Microvesicle
PAD	Peptidylarginine Deiminase
PHB	Prohibitin
STIM1	Stromal Interacting Molecule 1
TBS	Tris Buffered Saline
TMZ	Temozolomide
