# Supplementary material for: Peptidylarginine Deiminases Post-Translationally Deiminate Prohibitin and Modulate Extracellular Vesicle Release and MicroRNAs in Glioblastoma Multiforme
Source: Int J Mol Sci. 2018 Dec 28;20(1):103. doi: 10.3390/ijms20010103 (PMC6337164; doi:10.3390/ijms20010103)
Supplement: Supplementary file 1 [file ijms-20-00103-s001.pdf]

Table S1. Deiminated protein candidates identified by LC-MS/LS in LN18 and LN229 cells. Immunoprecipitated proteins from LN18 and LN229 cell protein extract, using the F95 pan-deimination antibody, were subjected to LC-MS/MS analysis and peak list files submitted to MASCOT; identified deiminated mitochondrial, nuclear, cytoskeletal, EV-related, stress-related and invadopodia-related proteins are shown. The m/z values, peptide sequences and scores are indicated.

Deiminated Target Proteins	GBM line	Symbol	m/z	Peptide sequence	Score [1]
Mitochondrial Proteins					
Prohibitin (PHB)	LN18	C9JW96 | C9JW96_HUMAN	531.2761	K.AAIISAEGDSK.A	124
			575.2974	R.FDAGELITQR.E	
	LN229	C9JW96 | C9JW96_HUMAN	531.2780	K.AAIISAEGDSK.A	55
Prohibitin-2 (PHB-2)	LN18	J3KPX7 | J3KPX7_HUMAN	605.8726	R.VLPSIVNEVLK.S	55
			862.4641	R.IPWFQYPIIYDIR.A	
	LN229	J3KPX7 | J3KPX7_HUMAN	450.2871	R.AQVSLLIR.R	282
			491.2478	R.EYTAAVEAK.Q	
			605.8754	R.VLPSIVNEVLK.S	
			608.3143	K.IVQAEGEAEAAK.M	
			618.6671	R.IGGVQQDTILAEGLHFR.I	
Stress-70 protein	LN18	P38646 | GRP75_HUMAN	451.2270	K.VLENAEGAR.T	635
			479.7500	K.LFEMAYK.K	
			489.7293	K.AMQDAEVSK.S	
			616.3336	R.QAASSLQQASLK.L	
			645.8422	K.VQQTVQDLFGR.A	
			681.3731	R.AQFEGIVTDLIR.R	
			723.8834	K.SDIGEVILVGGMTR.M	
			725.8597	R.TTPSVVAFTADGER.L	
			784.8870	R.QAVTNPNNTFYATK.R	
			823.4401	R.VINEPTAAALAYGLDK.S	
			619.6373	R.VEAVNMAEGIIHDTETK.M	
	LN229	P38646 | GRP75_HUMAN	616.3348	R.QAASSLQQASLK.L	444
			645.8431	K.VQQTVQDLFGR.A	
			681.3743	R.AQFEGIVTDLIR.R	
			723.8840	K.SDIGEVILVGGMTR.M	
			725.8611	R.TTPSVVAFTADGER.L	
ATP-synthase subunit alpha	LN18	P25705 | ATPA_HUMAN	430.7536	R.QMSLLLR.R	588
			513.7995	K.AVDSLVPIGR.G	
			586.3180	R.VVDALGNAIDGK.G	
			644.3478	K.HALIIYDDLSK.Q	
			658.8727	K.TSIAIDTIINQK.R	
			712.3372	K.TGTAEMSSILEER.I	

			518.5830	R.EAYPGDVFYLHSR.L	
			788.3942	R.ILGADTSVDLEETGR.V	
			812.9460	R.TGAIVDVPVGEELLGR.V	
	LN229	P25705 | ATPA_HUMAN	408.2346	R.ELIIGDR.Q	366
			430.7546	R.QMSLLLR.R	
			586.3190	R.VVDALGNAIDGK.G	
			658.8745	K.TSIAIDTIINQK.R	
			518.5842	R.EAYPGDVFYLHSR.L	
			788.3964	R.ILGADTSVDLEETGR.V	
			812.9473	R.TGAIVDVPVGEELLGR.V	
Cytochrome C1	LN18	P08574 | CY1_HUMAN	455.7251	K.DVCTFLR.W	346
			649.8550	R.WASEPEHDHR.K	
			421.8531	R.GLLSSLDHTSIR.R	
			760.8536	R.HLVGVCYTEDEAK.E	
			638.9818	K.LFDYFPKPYPNSEAAR.A	
			885.4209	R.HGGEDYVFSLLTGYCEPPTGVSLR.E	
	LN229	P08574 | CY1_HUMAN	455.7262	K.DVCTFLR.W	122
			433.5732	R.GLLSSLDHTSIR.R	
			885.4202	R.HGGEDYVFSLLTGYCEPPTGVSLR.E	
Histone Proteins					
Histone H2B	LN229	U3KQK0 | U3KQK0_HUMAN	408.7322	R.EIQTAVR.L	97
			872.4112	K.AMGIMNSFVNDIFER.I	
Histone H3	LN229	B4DEB1 | B4DEB1_HUMAN	416.2502	K.STELLIR.K	30
Histone H4	LN229	P62805 | H4_HUMAN	495.2927	K.VFLENVIR.D	100
			590.8132	R.ISGLIYEETR.G	
Histone deacetylase 1	LN18	Q13547 | HDAC1_HUMAN	474.7506	R.VMTVSFHK.Y	399
			579.7998	R.ISICSSDKR.I	
			533.2690	K.YYAVNYPLR.D	
			687.8176	K.YGEYFPGTGDLR.D	
			476.2243	R.SIRPDNMSEYSK.Q	
			681.9890	R.DGIDDESYEAIFKPVMSK.V	
			745.0198	K.LHISPSNMTNQNTNEYLEK.I	
			838.3850	K.VMEMFQPSAVVLQCGSDSLSGDR.L	
Histone deacetylase 2	LN229	H3BM24 | H3BM24_HUMAN	476.2258	R.SIRPDNMSEYSK.Q	32
Histone H1x	LN18	P38646 | GRP75_HUMAN	604.3365	K.YSQLVVETIR.R	47
	LN229	P38646 | GRP75_HUMAN	473.2543	K.AGGSAALSPSK.K	163
			604.3365	K.YSQLVVETIR.R	
Histone-binding protein	LN18	Q09028 | RBBP4_HUMAN	487.2760	K.TVALWDLR.N	177
			736.3688	K.TPSSDVLVFDYTK.H	
			602.6111	K.HPSKPDPSGECNPDLR.L	
Cytoskeletal/EV-related					
AHNAK	LN18	Q09666 | AHNK_HUMAN	451.2339	K.ADVDISGPK.I	50
GAPDH	LN18	P04406 | G3P_HUMAN	398.2117	K.LTGMAFR.V	792
			403.7098	K.VGVNGFGR.I	
			406.2086	K.LTGMAFR.V	

			415.2228	K.QASEGPLK.G	
			435.2568	K.VIPELNGK.L	
			401.2059	R.VVDLMAHMASK.E	
			706.3963	R.GALQNIIPASTGAAK.A	
			765.8984	R.VPTANVSVVDLTCR.L	
			807.4508	K.LVINGNPITIFQER.D	
			882.4014	K.LISWYDNEFGYSNR.V	
			917.4606	K.IISNASCTTNCLAPLAK.V	
			681.3666	K.LVINGNPITIFQERDPSK.	
			743.7035	R.VIISAPSADAPMFVMGVNHEK.Y	
			759.6815	K.WGDAGAEYVVESTGVFTTMEK.A	
	LN229	P04406|G3P_HUMAN	398.2130	K.LTGMAFR.V	881
			403.7110	K.VGVNGFGR.I	
			406.2095	K.LTGMAFR.V	
			435.2575	K.VIPELNGK.L	
			401.2070	R.VVDLMAHMASK.E	
			444.2208	R.VVDLMAHMASKE	
			706.3971	R.GALQNIIPASTGAAK.A	
			765.9000	R.VPTANVSVVDLTCR.L	
			510.9364	R.VPTANVSVVDLTCR	
			807.4537	K.LVINGNPITIFQER.D	
			807.9458	K.LVINGNPITIFQER.D	
			580.6500	R.DGRGALQNIIPASTGAAK.A	
			882.4041	K.LISWYDNEFGYSNR.V	
			588.6068	K.LISWYDNEFGYSNR.V	
			917.4614	K.IISNASCTTNCLAPLAK.V	
			611.9775	K.IISNASCTTNCLAPLAK.V	
			681.0412	K.LVINGNPITIFQERDPSK.I	
			681.3674	K.LVINGNPITIFQERDPSK.I	
			738.3725	R.VIISAPSADAPMFVMGVNHEK.Y	
			9.6839	K.WGDAGAEYVVESTGVFTTMEK.A	
			1139.0227	K.WGDAGAEYVVESTGVFTTMEK.A	
			1009.9774	K.GILGYTEHQVVSSDFNSDTHSSTFDAGAGIAL	
Vimentin	LN229	P08670|VIME_HUMAN	457.7319	R.SYVTTSTR.T	839
			512.2587	R.QQYESVAAK.N	
			530.7667	R.QVDQLTNDK.A	
			544.7711	R.QDVDNASLAR.L	
			547.2675	K.FADLSEAANR.N	
			585.3604	K.ILLAELEQLK.G	
			662.3127	R.EEAENTLQSFR.Q	
			714.8594	R.SLYASSPGGVYATR.	
			511.9561	R.KVESLQEEIAFLK.K	
			556.9518	R.ETNLDSLPLVDTHSK.R	
			709.3588	R.KVESLQEEIAFLK.K	
			729.6618	R.EMEENFAVEAANYQDTIGR.L	
			981.5101	K.LHEEEIQELQAQIQEQHVQIDVDVSKPDLTAA	
Filamin-A	LN18	Q60FE5|Q60FE5_HUMAN	494.7725	K.KGEITGEVR.M	966
			544.7852	R.TPCEEILVK.H	
			578.3112	K.IVGPSGAAVPCK.V	

			613.8287	R.AWGPGLEGGVVGK.S	
			613.8879	R.LIALLEVLSQK.K	
			656.8451	K.VEPGLGADNSVVR.F	
			713.8787	R.EAGAGGLAIAVEGPSK.A	
			717.8618	R.ANLPQSFQVDTSK.A	
			750.8782	K.DAGEGGLSLAIEGPSK.A	
			501.5928	R.FVPAEMGTHTVSVK.Y	
			785.9052	R.GAGTGGLGLAVEGPSEAK.M	
			530.2515	R.YAPSEAGLHEMDIR.Y	
			534.9257	K.YNEQHVPGSPFTAR.V	
			549.6287	K.TGVAVNKPAEFTVDAK.H	
			826.9316	K.VTAQGPGLEPSGNIANK.T	
			425.4830	R.TGVELGKPTHFTVNAK.A	
			605.6562	K.VAQPTITDNKDGTVTVR.Y	
			762.7270	K.YTPVQQGPVGVNVTYGGDPIPK.S	
			771.7021	R.SAGQGEVLVYVEDPAGHQEEAK.V	
			846.0536	R.YWPQEAGEYAVHVLCNSEDIR.L	
			899.1047	K.SADFVVEAIGDDVGTLGFSVEGPSQAK.I	
			982.5008	R.FGGEHVPNSPFQVTALAGDQPSVQPPLR.S	
	LN229	Q60FE5∣Q60FE5_HUMAN	395.7259	K.VYGPGVAK.T	804
			554.7907	K.RAEFTVETR.S	
			412.8945	K.KRAEFTVETR.S	
			708.3760	R.IANLQTDLSDGLR.L	
			717.3572	R.AYGPGIEPTGNMVK.K	
			521.2731	R.AYGPGIEPTGNMVKK.R	
			549.6299	K.TGVAVNKPAEFTVDAK.H	
			430.2316	R.AYGPGIEPTGNMVKKR.A	
			878.9210	K.SPFEVYVDKSQGDASK.V	
			586.2851	K.SPFEVYVDKSQGDASK.V	
			597.2811	K.ATCAPQHGAPGPGPADASK.V	
			605.6576	K.VAQPTITDNKDGTVTVR.Y	
			623.2861	R.SPYTVTVGQACNPSACR.A	
			515.2393	K.THEAEIVEGENHTYCIR.F	
			771.7033	R.SAGQGEVLVYVEDPAGHQEEAK.V	
			855.7354	K.AHEPTYFTVDCAEAGQGDVSIGIK.C	
			982.5020	R.FGGEHVPNSPFQVTALAGDQPSVQPPLR.S	
			603.9293	R.TFSVWYVPEVTGTHKVTVLFAGQHIAK.S	
Actin (cytoplasmic 1)	LN18	P60709∣ACTB_HUMAN	389.1800	K.CDVDIR.K	1221
			398.2388	K.IIAPPER.K	
			400.7702	K.RGILTLK.Y	
			488.7261	K.AGFAGDDAPR.A	
			507.7431	R.DLTDYLMK.I	
			566.7653	R.GYSFTTTAER.E	
			586.2863	R.HQGVMVGMGQK.D	
			589.3089	K.EITALAPSTMK.I	
			396.5251	K.DSYVGDEAQSK.R	
			599.7627	K.DSYVGDEAQSK.R	
			505.9197	K.IWHHTFYNELR.V	
			758.8536	K.QEYDESGPSIVHR.K	

			895.9474	K.SYELPDGQVITIGNER.F	
			977.5330	R.VAPEEHPVLLTEAPLNPK.A	
			1108.0349	K.DLYANTVLSGGTTMYPGIADR.M	
			781.7234	R.KDLYANTVLSGGTTMYPGIADR.M	
			1275.5862	K.LCYVALDFEQEMATAASSSSLEK.S	
			936.4388	K.EKLCYVALDFEQEMATAASSSSLEK.S	
			800.6543	R.TTGIVMDSGDGVTHTVPIYEGYALPHAILR.L	
			1077.8230	R.CPEALFQPSFLGMESCGIHETTFNSIMK.C	
	LN229	P60709|ACTB_HUMAN	389.1811	K.CDVDIR.K	
			398.2395	K.IIAPPER.K	
			400.7711	K.RGILTLK.Y	
			488.7273	K.AGFAGDDAPR.A	
			499.7463	R.DLTDYLMK.I	
			566.7664	R.GYSFTTTAER.E	
			586.2862	R.HQGVMVGMGQK.D	
			599.7645	K.DSYVGDEAQSK.R	
			400.2399	R.AVFPSIVGRPR.H	
			505.9210	K.IWHHTFYNELR.V	
			506.2395	K.QEYDESGPSIVHR.K	
			895.9484	K.SYELPDGQVITIGNER.F	
			652.0254	R.VAPEEHPVLLTEAPLNPK.A	
			739.0274	K.DLYANTVLSGGTTMYPGIADR.M	
			850.7290	K.LCYVALDFEQEMATAASSSSLEK.S	
			796.6578	R.TTGIVMDSGDGVTHTVPIYEGYALPHAILR.L	
Alpha-actinin-1	LN18	H9KV75|H9KV75_HUMAN	413.2252	R.LDHLAEK.F	446
			432.7431	K.ALDFIASK.G	
			405.5303	K.ASIHEAWTDGK.E	
			608.3379	K.LASDLLEWIR.R	
			693.8884	R.VGWEQLLTTIAR.T	
			711.3546	K.GYEEWLLNEIR.R	
			715.3835	R.TINEVENQILTR.D	
			856.4578	K.LLETIDQLYLEYAK.R	
	LN229	H9KV75|H9KV75_HUMAN	608.3406	K.LASDLLEWIR.R	229
			693.8905	R.VGWEQLLTTIAR.T	
			715.3848	R.TINEVENQILTR.D	
			780.4015	K.IDQLEGDHQLIQEALIFDNK.H	
Alpha-actinin-4	LN18	O43707|ACTN4_HUMAN	413.2252	R.LDHLAEK.F	858
			432.7431	K.ALDFIASK.G	
			447.7728	R.EAILAIHK.E	
			503.2541	K.TIQEMQQK.L	
			387.1936	K.STLPDADRER.E	
			405.5303	K.ASIHEAWTDGK.E	
			608.3379	K.LASDLLEWIR.R	
			442.5637	K.RDHALLEEQSK.Q	
			676.8143	K.GISQEQMQEFR.A	
			693.8884	R.VGWEQLLTTIAR.T	
			711.3546	K.GYEEWLLNEIR.R	
			715.3835	R.TINEVENQILTR.D	
			478.5765	R.LSNRPAFMPSEGK.M	

			516.9252	K.HRDYETATLSDIK.A	
			559.3053	K.VLAGDKNFITAEELR.R	
			871.4072	R.ETTDTDTADQVIASFK.V	
			606.2951	K.MLDAEDIVNTARPDEK.A	
			640.9803	K.AGTQIENIDEDFRDGLK.L	
			687.6559	K.VLAVNQENEHLMEDYEK.L	
	LN229	O43707 | ACTN4_HUMAN	608.3406	K.LASDLLEWIR.R	275
			442.5653	K.RDHALLEEQSK.Q	
			676.8174	K.GISQEQMQEFR.A	
			693.8905	R.VGWEQLLTTIAR.T	
			715.3848	R.TINEVENQILTR.D	
Tubulin alpha chain	LN18	F5H5D3 | F5H5D3_HUMAN	391.2062	R.LSVDYGK.K	1203
			444.2190	K.FDLMYAK.R	
			453.7144	R.EDMAALEK.D	
			455.2540	R.LSVDYGKK.S	
			508.2916	K.DVNAAIATIK.T	
			512.2263	K.EDAANNYAR.G	
			543.3119	K.EIIDLVLDR.I	
			625.2787	K.YMACCLLYR.G	
			460.9025	R.LDHKFDLMYAK.R	
			792.8769	R.SIQFVDWCPTGFK.V	
			851.4533	R.AVFVDLEPTVIDEVR.T	
			859.9415	R.NLDIERPTYTNLNR.L	
			878.9824	R.IHFPLATYAPVISAEK.A	
			912.9938	K.VGINYQPPTVVPGGDLAK.V	
			932.9526	R.AVCMLSNTTAIAEAWAR.L	
			1004.4476	K.TIGGGDDSFNTFFSETGAGK.H	
			1205.1050	R.FDGALNVDLTEFQTNLVPYPR.I	
			805.7378	R.QLFHPEQLITGKEDAANNYAR.G	
			917.4324	K.AYHEQLSVAEITNACFEPANQMVK.C	
			1079.4381	R.EDMAALEKDYEEVGVDSVEGEGEEEGEEY.-	
	LN229	F5H5D3 | F5H5D3_HUMAN	391.2077	R.LSVDYGK.K	884
			508.2920	K.DVNAAIATIK.T	
			512.2283	K.EDAANNYAR.G	
			543.3136	K.EIIDLVLDR.I	
			625.2792	K.YMACCLLYR.G	
			460.9036	R.LDHKFDLMYAK.R	
			470.9289	R.QLFHPEQLITGK.E	
			851.4562	R.AVFVDLEPTVIDEVR.T	
			573.6321	R.NLDIERPTYTNLNR.L	
			586.3269	R.IHFPLATYAPVISAEK.A	
			912.9976	K.VGINYQPPTVVPGGDLAK.V	
			1004.4490	K.TIGGGDDSFNTFFSETGAGK.H	
			803.7405	R.FDGALNVDLTEFQTNLVPYPR.I	
			922.1043	K.AYHEQLTVAEITNACFEPANQMVK.C	
Tubulin alpha-1B	LN18	P68363 | TBA1B_HUMAN	391.2062	R.LSVDYGK.K	1408
			444.2190	K.FDLMYAK.R	
			453.7144	R.EDMAALEK.D	
			455.2540	R.LSVDYGKK.S	

			508.2916	K.DVNAAIATIK.T	
			512.2263	K.EDAANNYAR.G	
			543.3119	K.EIIDLVLDR.I	
			625.2787	K.YMACCLLYR.G	
			460.9025	R.LDHKFDLMYAK.R	
			792.8769	R.SIQFVDWCPTGFK.V	
			851.4533	R.AVFVDLEPTVIDEVR.T	
			859.9415	R.NLDIERPTYTNLNR.L	
			878.9824	R.IHFPLATYAPVISAEK.A	
			912.9938	K.VGINYQPPTVVPGGDLAK.V	
			932.9526	R.AVCMLSNTTAIAEAWAR.L	
			1004.4476	K.TIGGGDDSFNTFFSETGAGK.H	
			777.3413	R.AFVHWYVGEGMEEGEFSEAR.E	
			1205.1050	R.FDGALNVDLTEFQTNLVPYPR.I	
			805.7378	R.QLFHPEQLITGKEDAANNYAR.G	
			917.4324	K.AYHEQLSVAEITNACFEPANQMVK.C	
			1079.4381	R.EDMAALEKDYEEVGVDSVEGEGEEEGEEY.-	
	LN229	P68363|TBA1B_HUMAN	388.2092	R.GHYTIGK.E	1039
			391.2077	R.LSVDYGK.K	
			508.2920	K.DVNAAIATIK.T	
			512.2283	K.EDAANNYAR.G	
			543.3136	K.EIIDLVLDR.I	
			625.2792	K.YMACCLLYR.G	
			460.9036	R.LDHKFDLMYAK.R	
			470.9289	R.QLFHPEQLITGK.E	
			792.8789	R.SIQFVDWCPTGFK.V	
			851.4562	R.AVFVDLEPTVIDEVR.T	
			573.6321	R.NLDIERPTYTNLNR.L	
			586.3269	R.IHFPLATYAPVISAEK.A	
			912.9976	K.VGINYQPPTVVPGGDLAK.V	
			932.9543	R.AVCMLSNTTAIAEAWAR.L	
			1004.4490	K.TIGGGDDSFNTFFSETGAGK.H	
			803.7405	R.FDGALNVDLTEFQTNLVPYPR.I	
			917.4354	K.AYHEQLSVAEITNACFEPANQMVK.C	
Tubulin alpha-4A	LN18	P68366|TBA4A_HUMAN	391.2062	R.LSVDYGK.K	1077
			444.2190	K.FDLMYAK.R	
			453.7144	R.EDMAALEK.D	
			455.2540	R.LSVDYGKK.S	
			512.2263	K.EDAANNYAR.G	
			535.2969	K.EIIDPVLDR.I	
			625.2787	K.YMACCLLYR.G	
			460.9025	R.LDHKFDLMYAK.R	
			792.8769	R.SIQFVDWCPTGFK.V	
			859.9415	R.NLDIERPTYTNLNR.L	
			878.9824	R.IHFPLATYAPVISAEK.A	
			912.9938	K.VGINYQPPTVVPGGDLAK.V	
			932.9526	R.AVCMLSNTTAIAEAWAR.L	
			777.3413	R.AFVHWYVGEGMEEGEFSEAR.E	
			1205.1050	R.FDGALNVDLTEFQTNLVPYPR.I	

			805.7378	R.QLFHPEQLITGKEDAANNYAR.G	
			917.4324	K.AYHEQLSVAEITNACFEPANQMVK.C	
Tubulin beta	LN18	P07437丨TBB5_HUMAN	514.7613	K.TAVCDIPPR.G	1364
			520.2991	R.YLTVAAVFR.G	
			533.2160	K.NMMAACDPR.H	
			539.2678	K.IREEYPDR.I	
			565.7998	R.FPGQLNADLR.K	
			580.3170	K.LAVNMVPFPR.L	
			615.3007	R.ISEQFTAMFR.R	
			420.2336	R.FPGQLNADLRK.L	
			651.3197	R.ISVYYNEATGGK.Y	
			660.3536	R.IMNTFSVVPSPK.V	
			723.8456	K.EVDEQMLNVQNK.N	
			808.4189	R.AILVDLEPGTMDSVR.S	
			540.9487	R.LHFFMPGFAPLTSR.G	
			830.4487	R.ALTVPELTQQVFDAK.N	
			848.9175	K.NSSYFVEWIPNNVK.T	
			911.9622	R.EIVHIQAGQCGNQIGAK.F	
			935.4907	K.MAVTFIGNSTAIQELFK.R	
			979.9920	K.GHYTEGAELVDSVLDVVR.K	
			933.7769	R.SGPFGQIFRPDNFVFGQSGAGNNWAK.G	
			1034.8059	K.FWEVISDEHGIDPTGTYHGDSDLQLDR.I	
			1104.5138	K.EAESCDCLQGFQLTHSLGGGTGSGMGTLLISK	
			1149.2805	K.VSDTVVEPYNATLSVHQLVENTDETYCIDNE	
	LN229	P07437丨TBB5_HUMAN	514.7639	K.TAVCDIPPR.G	1028
			520.3002	R.YLTVAAVFR.G	
			533.2171	K.NMMAACDPR.H	
			539.2700	K.IREEYPDR.I	
			565.8015	R.FPGQLNADLR.K	
			572.3225	K.LAVNMVPFPR.L	
			615.3035	R.ISEQFTAMFR.R	
			424.5810	R.KLAVNMVPFPR.L	
			651.3218	R.ISVYYNEATGGK.Y	
			660.3549	R.IMNTFSVVPSPK.V	
			816.4207	R.AILVDLEPGTMDSVR.S	
			830.4508	R.ALTVPELTQQVFDAK.N	
			608.3121	R.EIVHIQAGQCGNQIGAK.F	
			935.4925	K.MAVTFIGNSTAIQELFK.R	
			653.6652	K.GHYTEGAELVDSVLDVVR.K	
			776.3545	K.FWEVISDEHGIDPTGTYHGDSDLQLDR.I	
			1104.5167	K.EAESCDCLQGFQLTHSLGGGTGSGMGTLLISK	
			1149.2822	K.VSDTVVEPYNATLSVHQLVENTDETYCIDNE	
Tubulin beta-3	LN18	Q13509丨TBB3_HUMAN	533.2160	K.NMMAACDPR.H	860
			565.7998	R.FPGQLNADLR.K	
			580.3170	K.LAVNMVPFPR.L	
			615.3007	R.ISEQFTAMFR.R	
			420.2336	R.FPGQLNADLRK.L	
			660.3536	R.IMNTFSVVPSPK.V	
			695.8445	K.EVDEQMLAIQSK.N	

			808.4189	R.AILVDLEPGTMDSVR.S	
			846.4347	R.ALTVPELTQQMFDAK.N	
			848.9175	K.NSSYFVEWIPNNVK.V	
			911.9622	R.EIVHIQAGQCGNQIGAK.F	
			979.9920	K.GHYTEGAELVDSVLDVVR.K	
			1026.4730	K.FWEVISDEHGIDPSGNYVGDSDLQLER.I	
	LN229	Q13509|TBB3_HUMAN	533.2171	K.NMMAACDPR.H	644
			565.8015	R.FPGQLNADLR.K	
			572.3225	K.LAVNMVPFPR.L	
			615.3035	R.ISEQFTAMFR.R	
			424.5810	R.KLAVNMVPFPR.L	
			660.3549	R.IMNTFSVVPSPK.V	
			695.8459	K.EVDEQMLAIQSK.N	
			466.5609	R.ISVYYNEASSHK.Y	
			816.4207	R.AILVDLEPGTMDSVR.S	
			846.4385	R.ALTVPELTQQMFDAK.N	
			608.3121	R.EIVHIQAGQCGNQIGAK.F	
			653.6652	K.GHYTEGAELVDSVLDVVR.K	
Tubulin beta-4B	LN18	P68371|TBB4B_HUMAN	514.7613	K.TAVCDIPPR.G	1208
			520.2991	R.YLTVAAVFR.G	
			533.2160	K.NMMAACDPR.H	
			539.2678	K.IREEYPDR.I	
			565.7998	R.FPGQLNADLR.K	
			580.3170	K.LAVNMVPFPR.L	
			615.3007	R.ISEQFTAMFR.R	
			420.2336	R.FPGQLNADLRK.L	
			660.3536	R.IMNTFSVVPSPK.V	
			664.8255	R.INVYYNEATGGK.Y	
			723.8456	K.EVDEQMLNVQNK.N	
			801.4119	R.AVLVDLEPGTMDSVR.S	
			846.4347	R.ALTVPELTQQMFDAK.N	
			848.9175	K.NSSYFVEWIPNNVK.T	
			911.9622	R.EIVHLQAGQCGNQIGAK.F	
			979.9920	K.GHYTEGAELVDSVLDVVR.K	
			933.7769	R.SGPFGQIFRPDNFVFGQSGAGNNWAK.G	
			1104.5138	K.EAESCDCLQGFQLTHSLGGGTGSGMGTLLISK	
	LN229	P68371|TBB4B_HUMAN	514.7639	K.TAVCDIPPR.G	858
			520.3002	R.YLTVAAVFR.G	
			533.2171	K.NMMAACDPR.H	
			539.2700	K.IREEYPDR.I	
			565.8015	R.FPGQLNADLR.K	
			572.3225	K.LAVNMVPFPR.L	
			615.3035	R.ISEQFTAMFR.R	
			424.5810	R.KLAVNMVPFPR.L	
			660.3549	R.IMNTFSVVPSPK.V	
			664.8265	R.INVYYNEATGGK.Y	
			846.4385	R.ALTVPELTQQMFDAK.N	
			608.3121	R.EIVHLQAGQCGNQIGAK.F	
			653.6652	K.GHYTEGAELVDSVLDVVR.K	

			779.8595	K.FWEVISDEHGIDPTGTYHGDSDLQLER.I	
			1104.5167	K.EAESCDCLQGFQLTHSLGGGTGSGMGTLLISK	
Tubulin beta-6	LN18	Q9BUF5lTBB6_HUMAN	533.2160	R.NMMAACDPR.H	553
			565.7998	R.FPGQLNADLR.K	
			580.3170	K.LAVNMVPFPR.L	
			608.2931	R.ISEQFSAMFR.R	
			420.2336	R.FPGQLNADLRK.L	
			695.8445	K.EVDEQMLAIQSK.N	
			540.9487	R.LHFFMPGFAPLTSR.G	
			848.9175	K.NSSYFVEWIPNNVK.V	
			648.3321	K.GHYTEGAELVDAVLDVVR.K	
			987.4709	K.FWEVISDEHGIDPAGGYVGDSALQLER.I	
			1149.2805	K.VSDTVVEPYNATLSVHQLVENTDETYCIDNE	
Stress-related					
GRP78	LN18	P11021lGRP78_HUMAN	459.7413	K.VLEDSDLK.K	900
			493.7597	R.LTPEEIER.M	
			537.7788	K.ITITNDQNR.L	
			596.3193	K.VYEGERPLTK.D	
			609.3172	K.DAGTIAGLNVMR.I	
			414.2115	K.MKETAEAYLGK.K	
			658.8211	R.NELESYAYSLK.N	
			699.3958	K.ELEEIVQPIISK.L	
			730.8839	K.SDIDEIVLVGGSTR.I	
			768.9004	K.TFAPEEISAMVLTK.M	
			783.8927	R.ITPSYVAFTPEGER.L	
			830.4487	R.IINEPTAAAIAYGLDK.R	
			839.4035	K.NQLTSNPENTVFDAK.R	
			658.9720	K.IEWLESHQDADIEDFK.A	
			1082.9957	R.IEIESFYEGEDFSETLTR.A	
	LN229	P11021lGRP78_HUMAN	459.7419	K.VLEDSDLK.K	
			493.7611	R.LTPEEIER.M	
			537.7805	K.ITITNDQNR.L	
			596.3223	K.VYEGERPLTK.D	
			404.5279	K.EFFNGKEPSR.G	
			609.3186	K.DAGTIAGLNVMR.I	
			614.8174	R.VEIIANDQGNR.I	
			414.2129	K.MKETAEAYLGK.K	
			657.3133	K.FEELNMDLFR.S	
			658.8223	R.NELESYAYSLK.N	
			730.8842	K.SDIDEIVLVGGSTR.I	
			504.9229	R.AKFEELNMDLFR.S	
			768.9021	K.TFAPEEISAMVLTK.M	
			783.8944	R.ITPSYVAFTPEGER.L	
			830.4517	R.IINEPTAAAIAYGLDK.R	
			839.4060	K.NQLTSNPENTVFDAK.R	
			918.9691	K.SQIFSTASDNQPTVTIK.V	
			1082.9978	R.IEIESFYEGEDFSETLTR.A	

G3P	LN18 /LN229	P04406∣G3P_HUMAN	*See GAPDH above*		792 /881
GBM-invasion related					
STIM1L	LN18	G0XQ39∣G0XQ39_HUMAN	579.8253	K.QALSEVTAALR.E	53
	LN229	G0XQ39∣G0XQ39_HUMAN	579.8273	K.QALSEVTAALR.E	130
			56.9416	K.ALDTVLFGPPLLTR.H	
Moesin	LN18	P26038∣MOES_HUMAN	417.2405	K.ESPLLFK.F	196
			488.7751	K.QLFDQVVK.T	
			591.7991	K.APDFVFYAPR.L	
			617.2883	K.ESEAVEWQQK.A	
			491.5996	R.RKPDTIEVQQMK.A	
			694.6724	K.FYPEDVSEELIQDITQR.L	
Annexin A1	LN229	P04083∣ANXA1_HUMAN	454.7273	R.ALYEAGER.R	223
			775.9124	K.GTDVNVFNTILTTR.S	
			851.9451	K.GLGTDEDTLIEILASR.T	
			786.0572	K.GGPGSAVSPYPTFNPSSDVAALHK.A	
Cathepsin D	LN18	A0A1B0GV23∣A0A1B0GV23_ HUMAN	440.2315	R.QVFGEATK.Q	30/35
	LN229	A0A1B0GV23∣A0A1B0GV23_ HUMAN	521.8289	K.VSTLPAITLK.L	
GAPDH	LN18/ LN229	P04406∣G3P_HUMAN	*see GAPDH above*		792/ 881
Integrin beta-1	LN229	Q5T3E6∣Q5T3E6_HUMAN	554.2880	R.SGEPQTFTLK.F	108
			673.7234	K.LKPEDITQIQPQQLVLR.L	

[1] Ions score is −10*Log(P), where P is the probability that the observed match is a random event. Individual ions scores >28 indicate identity or extensive homology (p < 0.05). Protein scores are derived from ions scores as a non-probabilistic basis for ranking protein hits. Cut-off was set at Ions score 20.

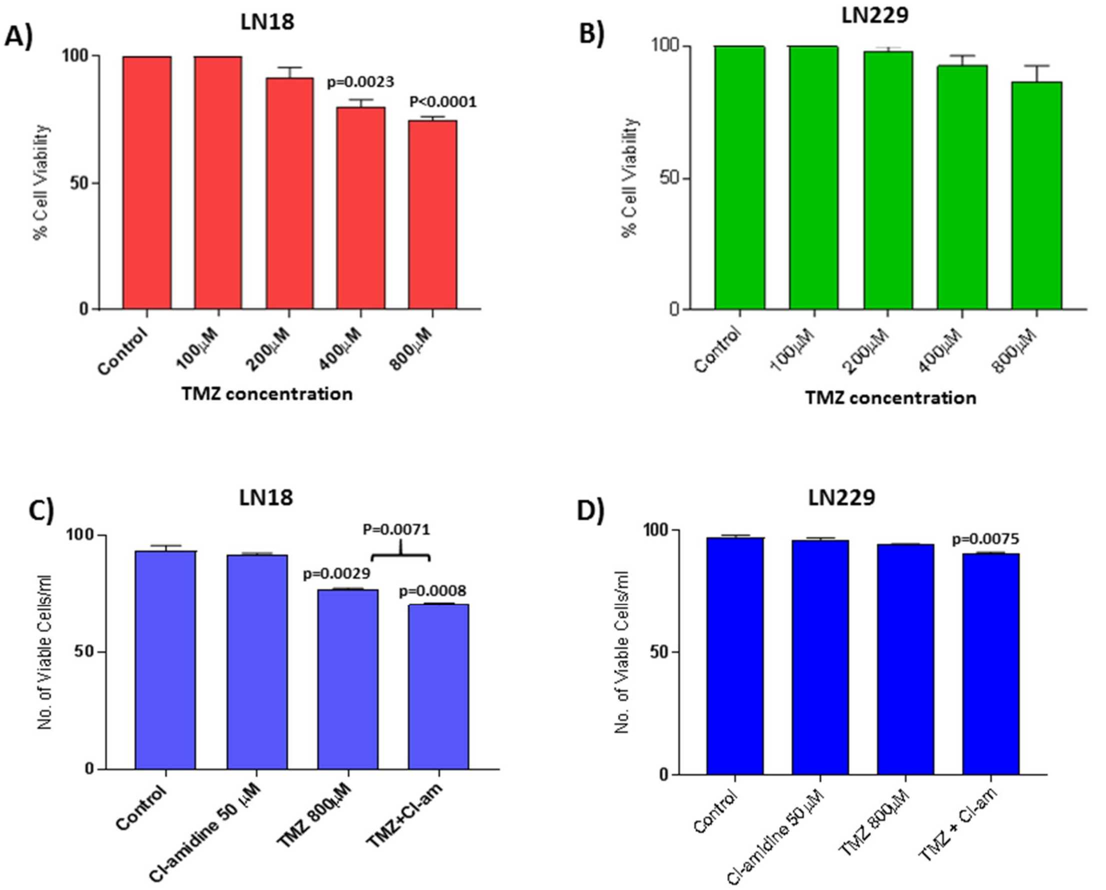

Figure S1. GBM cell viability after 1 h incubation with Cl-amidine, TMZ and Cl-amidine in combination with TMZ. (**A**,**B**) Viability of GBM cells LN18 and LN229 was assessed after 1 h incubation with 100–800 μM TMZ; (**A**) LN18 cells showed a 15% decrease in viability at 400 μM and 23% decrease in viability at 800 μM TMZ respectively; (**B**) LN229 showed non-significant 5% decrease in cell viability in the presence of 800 μM TMZ, compared to DMSO treated control cells. (**C**) LN18 viability was not significantly affected by Cl-amidine alone, while TMZ-Cl-amidine combinatory treatment caused a 24.7% reduction in cell viability. (**D**) LN229 viability was not significantly affected by Cl-amidine alone but was reduced by 7.22% after 1 h incubation with TMZ-Cl-amidine combinatory treatment.
